# Dissecting the relationship between childhood abuse, personality traits and genetic markers in eating disorders using a discordant sister-pair design

**DOI:** 10.1186/2050-2974-1-S1-O69

**Published:** 2013-11-14

**Authors:** Isabel Krug, Matthew Fuller-Tyszkiewicz, Nadia Micali, Emma Taborelli, Marija Anderluh, Fernando Fernandez-Aranda, Kate Tchanturia, Andreas Karwautz, Gudrun Wagner, David Collier, Janet Treasure

**Affiliations:** 1 University of Melbourne, Australia; 2 Deakin University, Australia; 3 University College London, UK; 4 University Medical Centre Ljubliana, Slovenia; 5 University Hospital Bellvitg, Spain; 6 King’s College London, UK; 7 University of Vienna, Austria

## Objective

To evaluate the profile of genetic markers, childhood abuse and personality in eating disorders (EDs) and compare this to the profiles found in their healthy sisters. Gene environment interactions (G*E) were also assessed for sexual abuse and various candidate genes.

## Method

Participants were 147 discordant sister pairs (total of 297 participants) for EDs. The semi-structured EATATE interview [designed to assess ED symptomatology and obsessive-compulsive personality traits (OCPD)], the Temperament and Character Inventory (TCI-R) and the Oxford Risk Factor Interview (ORFI) were used. DNA was also collected and three candidate genes (5-HT2A, BDNF and 5-HTTLPR) were genotyped.

## Results

ED patients presented with significantly more adulthood OCPD traits, higher levels of harm-avoidance and lower levels of self-directedness and cooperativeness than their healthy sisters (*p*<.01).Compared to the sisters, the ED group revealed more significant associations between different personality measures and the candidate genes 5-HTTLPR and 5-HT2A (*p*<.05). Sexual abuse also occurred more commonly in the ED group compared to the sisters. The only personality traits associated with sexual abuse were adulthood OCPD traits (p <.05). Finally, for the G*E interaction effects, even though no significant results were obtained for either of the 3 candidate genes, the likelihood of an ED increased by roughly 4.5 times for those patients with the presence of the LS-5-HTTLPR genotype and sexual abuse.

## Conclusions

Our findings indicate that EDs are complex genetic disorders of multi-factorial aetiology, where liability may result from the accumulation of psychosocial risk factors that trigger the illness in genetically vulnerable individuals.

This abstract was presented in the **Understanding and Treating Eating Pathology** stream of the 2013 ANZAED Conference.

